# Case study of monozygotic triplets with identical pineal cysts. Is pineal cyst hereditary condition?

**DOI:** 10.1007/s00701-026-06966-5

**Published:** 2026-06-24

**Authors:** Neevya Balasubramaniam, Zdeněk Musil, Kateřina Chroboková, Michael Tonhajzer, Tomáš Moravec, David Netuka, Vladimír Beneš, Martin Májovský

**Affiliations:** 1 Department of Neurology and Neurosurgery, McGill University, Montreal, Canada; 2 Institute of Biology and Medical Genetics, First Faculty of Medicine, Charles University and General University Hospital, Prague, Czech Republic; 3 Department of Anatomy, Third Faculty of Medicine, Charles University, Prague, Czech Republic; 4 Third Faculty of Medicine, Charles University, Prague, Czech Republic; 5 Faculty of Mechanical Engineering, Czech Technical University, 160 00 Prague, Czech Republic; 6 Department of Neurosurgery and Neurooncology, First Faculty of Medicine, Charles University and Military University Hospital, Prague, Czech Republic

**Keywords:** Pineal cyst, Monozygotic triplets, Hereditary predisposition, Whole-exome sequencing, Familial clustering, HERC2

## Abstract

**Background:**

Pineal cysts are common incidental findings of uncertain etiology, and the potential contribution of genetic factors to their development remains poorly understood. This study aimed to investigate the possibility of a hereditary predisposition to pineal cysts by examining a unique case of identical pineal cysts in a set of monozygotic triplets and by surveying familial occurrence of pineal cysts within an institutional patient database.

**Methods:**

A retrospective case study was conducted on 21-year-old monozygotic male triplets, each identified with a pineal gland cyst. All three underwent brain MRI with dedicated evaluation of the pineal region, independently reviewed by two board-certified neuroradiologists. One symptomatic triplet (T1) underwent surgical resection; peripheral blood and resected cyst tissue were subjected to whole-exome sequencing using an Agilent/Illumina NextSeq platform with variant analysis against the GRCh38 reference genome. Additionally, an institutional database of 243 patients with pineal cysts was reviewed for familial clustering.

**Results:**

All three triplets presented with morphologically identical 14 mm pineal cysts, despite variable clinical presentations. WES identified a germline *HERC2* nonsense variant (c.7114G > T, p.Glu2372Ter) in peripheral blood and a somatic *INO80E* frameshift mutation (c.42del) in cyst tissue, suggesting a possible two-hit model. Database review revealed eight additional individuals across four unrelated families with pineal cysts, all first-degree relatives, with identical cyst sizes noted in three of the four families.

**Conclusion:**

These findings provide novel evidence supporting a potential hereditary component in pineal cyst formation. While causality cannot be established from this case series alone, the observed familial clustering and identified genetic variants suggest that genetic factors may contribute to pineal cyst formation in selected cases and merit further investigation as additional familial or genetically characterized cases become available.

## Introduction

The pineal gland plays an important role in regulating sleep cycle and maintain the circadian rhythms [4] by producing melatonin [13]. Pineal cysts are benign glial cyst [17] that forms in the pineal gland, typically asymptomatic [23] and are often found incidentally. Some cases may be symptomatic, with presentations including headaches, hydrocephalus, oculomotor disorders, or rarely death [1, 6]. In the literature, there are previous autopsy series in the literature reporting up to 40% [16, 26, 27] while MRI studies reported the prevalence to range from 0.5–4% for cysts above 5 mm, with a more recent study showing 37.5% for size-independent cysts [28]. Thus, the true prevalence in the healthy population remains under discussion but pineal gland cysts have female predominance [2, 3].neration of pineal gland or congenital origins including remnant of pineal diverticulum in early stages [19] or enlarged cavum pineale [9], necrosis or hemorrhage of the gland in fetal stages [22] or due to ischemic damage to glial plate [20].

The genetic contribution to pineal cyst formation remains poorly defined. To date, Yan et al. have provided the only systematic sequencing analysis specifically focused on pineal cysts, using whole-exome sequencing of germline DNA from affected individuals and somatic DNA from resected cyst tissue; their findings suggested genetic heterogeneity and possible germline-somatic contributions, but did not identify a single recurrent causal alteration [29]. Additional rare syndromic observations, including pineal cysts reported in a patient with de novo trisomy 20p and in UBE2A deficiency syndrome, further suggest that pineal cysts may occasionally occur in the context of broader genomic or neurodevelopmental disorders, although these isolated associations do not establish causality [7, 10]. Indirect genetic support also comes from retinoblastoma cohorts, in which cystic pineal gland findings have been described in the setting of RB1-associated disease and familial retinoblastoma, suggesting that pathways involved in pineal-region development and oncogenesis may partially overlap with benign cystic phenotypes [15]. However, this association remains speculative and does not imply that pineal cysts are neoplastic.

Our case study is the first to our knowledge exploring the case of identical pineal cysts in a set of monozygotic triplets to further raise questions regarding the role of genetics in pineal cysts and emphasize the need for further research.

## Methods and materials

We conducted a case study of 21-year-old monozygotic male triplets (patient T1, T2 and T3), each found to have a pineal gland cyst. Clinical and demographic data were obtained through a retrospective review of medical records and structured interviews with the patients and their mother. A detailed birth history was collected, including gestational age, mode of delivery, and any complications during pregnancy or the neonatal period. All three siblings were born at term via scheduled, uncomplicated cesarean section, with no reported perinatal or neonatal complications.

All three patients underwent brain magnetic resonance imaging (MRI) with dedicated evaluation of the pineal region. T1-weighted and T2-weighted sequences, including contrast-enhanced imaging, were used to assess cyst size, morphology, and signal characteristics. Imaging studies were independently reviewed by two board-certified neuroradiologists.

Patient T1 underwent surgical resection of the pineal cyst. Peripheral blood and resected pineal cyst tissue from this patient were obtained for genetic analysis by whole-exome sequencing (WES). DNA was extracted from both samples using the QIAamp DNA Mini Kit (Qiagen, USA), and DNA quality was assessed using a NanoDrop™ 2000/2000c spectrophotometer (Thermo Scientific). Whole-exome sequencing was performed on DNA isolated from peripheral blood and pineal cyst tissue using an Agilent library preparation kit and sequenced on an Illumina NextSeq platform. Sequencing data were analyzed using GENOVESA v14.1 software (BIOXSYS, Czech Republic) and Integrative Genomics Viewer (IGV) version 2.18.4. Raw sequencing reads were quality-checked, trimmed if necessary and aligned to the GRCh38 reference genome. Variant calling was performed separately for both samples. Variants present in the pineal cyst sample were compared with the matched blood DNA to distinguish somatic changes from germline variants. The final filtered variant set was annotated using public genomic databases (GRCh38, Hg38) and prioritized according to predicted functional impact, population frequency, and relevance to pineal cyst-associated pathways.

In addition, we performed a retrospective review of our institutional database to identify other patients and families with pineal cysts. Available clinical data, imaging findings, and genetic testing results from these individuals were reviewed to identify potential familial patterns or shared characteristics suggestive of a hereditary component.

The study was approved by the institutional research ethics board (no. 108/18–53/2023). Written informed consent was obtained from all participants prior to inclusion in the study.

## Results

### Monozygotic triplets

**Fig. 1:**
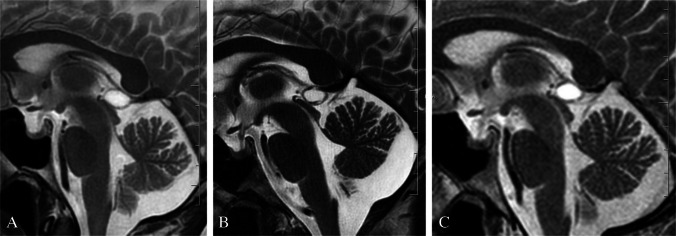
MRI of triplets with pineal cysts. **A** Sagittal T2-weigthed image of Triplet 1. **B** Sagittal T2-weigthed image of Triplet 2. **C** Sagittal T2-weigthed image of Triplet 3

The monozygotic triplets were of Middle Eastern origin and were delivered via a scheduled, uncomplicated cesarean section. There was no history of perinatal hypoxia, significant comorbidities, or other relevant medical history. All three siblings were found to have identical pineal gland cysts measuring 14 mm, which appeared as hyperintense thin-walled cystic lesions on T2-weighted MRI in the pineal region (Fig. 1). In addition, all three subjects showed similar enlargement of the cisterna magna and retrocerebellar subarachnoid space. Despite identical radiological findings, the clinical presentation varied among the three subjects.

Patient T1 presented with headache, retro-orbital pressure, gait instability, photophobia, and sleep disturbances. Contrast-enhanced MRI demonstrated a 14-mm pineal cyst with a tectum-splenium/cyst ratio of 0.88, consistent with previously described imaging biomarkers associated with central venous hypertension in symptomatic pineal cysts [12]. The patient underwent surgical treatment via a supracerebellar infratentorial approach in the sitting position, achieving planned subtotal cyst resection. The postoperative course was uneventful. At three-year follow-up, the patient reported partial improvement in symptoms, particularly headache and photophobia.

Patient T2 presented with isolated photophobia and has been managed conservatively. Patient T3 was asymptomatic, with the pineal cyst identified incidentally on imaging, and has likewise been followed without surgical intervention.

### Genetic analysis

Whole-exome sequencing of patient T1, followed by variant filtering using all abovementioned criteria, identified a germline HERC2 nonsense variant, c.7114G > T (p.Glu2372Ter), in peripheral blood and a somatic INO80E frameshift mutation, c.42del, in the pineal cyst specimen.

### Other cases of familial occurrence of pineal cysts

**Fig. 2:**
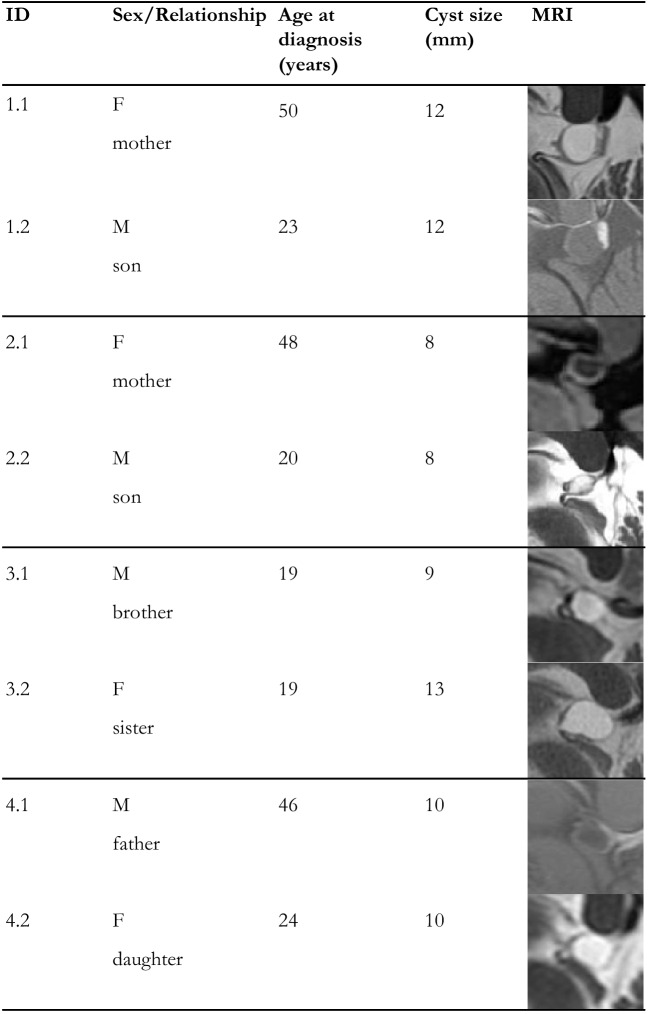
Demonstration of four families with pineal cysts

In addition to the triplet case, a review of our institutional database of 243 patients with pineal cysts identified eight additional affected individuals from four separate families (Fig. 2). All were first-degree relatives. One family included a 50-year-old woman (patient 1.1) who presented with severe headache and was found to have a 12-mm pineal cyst that was subsequently resected; her 23-year-old son (patient 1.2) was also found to have an incidentally detected pineal cyst of identical size.

Another family consisted of a 48-year-old mother (patient 2.1) and her 20-year-old son (patient 2.2), both diagnosed with pineal cysts measuring 8 mm. The mother initially presented with blurred vision and was diagnosed first; her son later developed similar symptoms and was subsequently diagnosed with a pineal cyst.

A third family included a 19-year-old brother (patient 3.1) and sister (patient 3.2) who were both diagnosed with pineal cysts measuring 9 mm and 13 mm, respectively. The sister presented with migraine headaches and was managed conservatively, whereas the brother was asymptomatic, and the cyst was discovered incidentally.

Finally, a father (patient 4.1) and daughter (patient 4.2) were diagnosed at 46 and 26 years of age, respectively, each with a 10-mm pineal cyst. The father was undergoing evaluation and treatment for a convexity meningioma, and the pineal cyst was an incidental finding. The daughter was evaluated after a single episode of headache, and the pineal cyst was considered asymptomatic. Notably, in three of the four families identified, the affected relatives had pineal cysts of identical size.

## Discussion

### Familial occurrence of pineal cysts

The present study provides evidence supporting a potential genetic contribution to the development of pineal cysts. The presence of morphologically identical cysts in monozygotic triplets who share the same genetic background and were exposed to similar intrauterine and early-life environments strongly suggests a heritable predisposition. Although environmental factors cannot be excluded, the remarkable similarity in cyst size, morphology, and anatomical location among the three siblings highlights the likely role of underlying genetic factors in cyst formation.

This hypothesis is further supported by the identification of eight additional affected individuals from four separate families in our institutional database. In several of these families, relatives were found to have pineal cysts of identical size, suggesting possible familial clustering. Genetic sequencing performed in the triplet case revealed potentially relevant variants, raising the possibility of a shared genetic susceptibility. Together, these findings support the concept of a hereditary component and raise the possibility that specific genetic variants may predispose certain individuals to developing pineal cysts. If confirmed in larger cohorts, this could improve risk stratification and inform future diagnostic and therapeutic strategies for patients with a family history of pineal cysts.

Our findings are in line with prior literature proposing a possible genetic etiology for pineal cysts [7, 10, 12, 15, 29]. Several studies have suggested familial tendencies or increased concordance rates among monozygotic twins with central nervous system anomalies, but the specific genes involved remain largely elusive. The unique presentation of three identical cysts in monozygotic triplets offers novel support for a genetic influence and represents a rare addition to the existing literature. Interestingly, Lannon et al. also addressed the possibility of a genetic etiology in colloid cysts, based on a case report of monozygotic twins [21].

### Genetics of pineal cyst

**Fig. 3:**
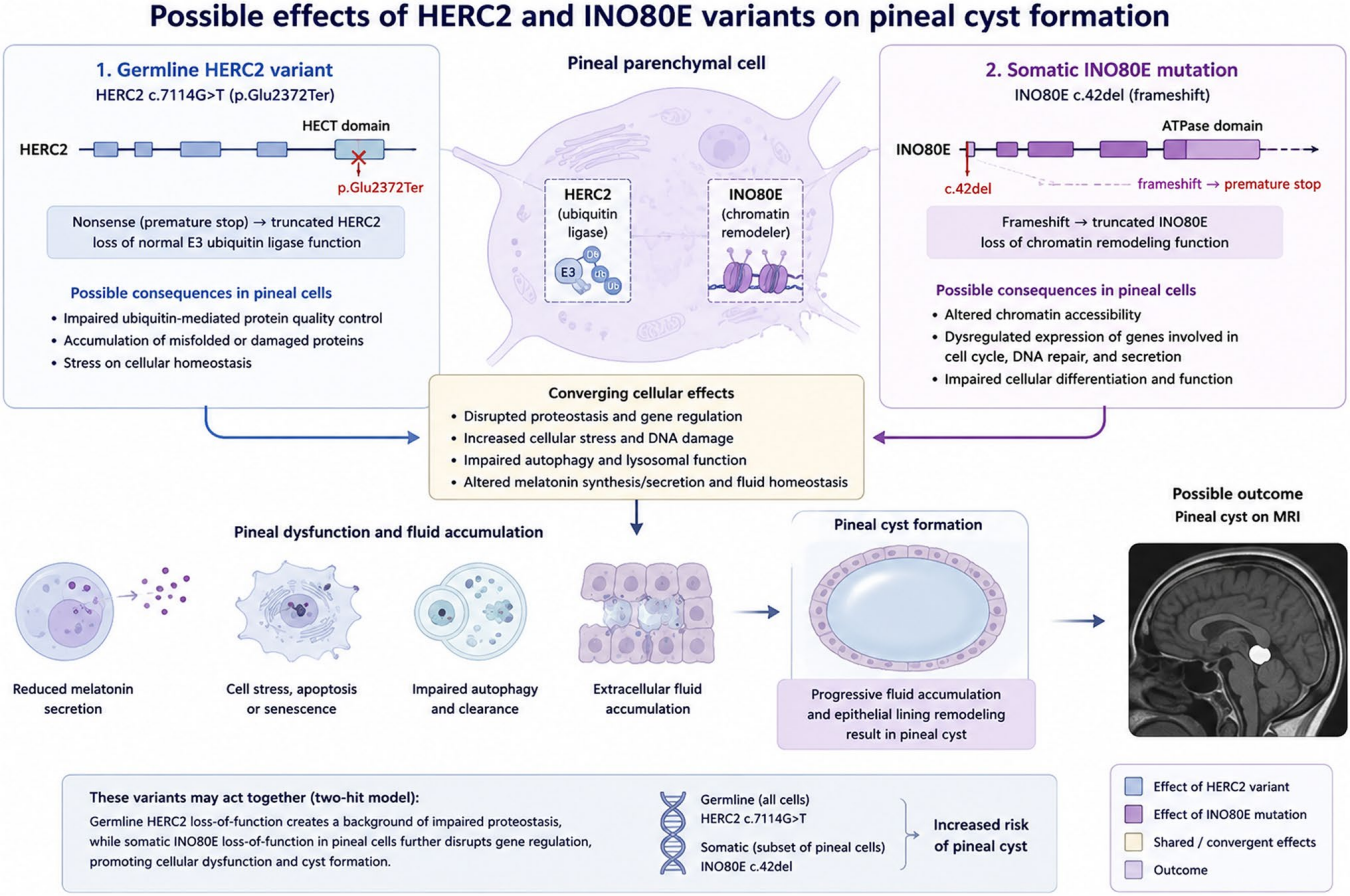
Possible effects of HERC2 and INO80E variants on PC formation

In present study, genetic analysis of our patient T1 identified a germline HERC2 c.7114G > T (p.Glu2372Ter) variant and a somatic INO80E c.42del frameshift mutation. HERC2 (HECT and RLD domain–containing E3 ubiquitin protein ligase 2) encodes a very large (~ 4,834 amino acid) E3 ubiquitin ligase that contains multiple RCC1-like (RLD) domains and a C-terminal HECT domain. The gene is widely expressed, with particularly high expression in the brain, and is involved in several cellular processes relevant to neural development and cellular regulation [18, 25]. HERC2 p.Glu2372Ter variant could represent potential germline “first hit” in the pineal cyst evolution, this variant can be found in any body cells. A possible somatic “second hit” might be the INO80E c.42del mutation. INO80E encodes a subunit of the ATP-dependent INO80 chromatin-remodeling complex, which plays an important role in nucleosome positioning, replication fork restart, and DNA repair [8] (see Fig. 3).

The proposed cooperation between HERC2 and INO80E is conceptually consistent with several observations. Whole-exome sequencing data from pineal cysts support a multi-hit model involving germline predisposition followed by somatic alterations that affect gene expression and chromatin biology [29]. HERC2 is a brain-expressed E3 ligase with established roles in neural development, and E3 ligases more broadly are increasingly recognized as key regulators of neurodevelopment and brain disease [24]. INO80E is involved in chromatin remodeling, DNA replication, and DNA repair, and somatic frameshift mutations in this gene have been reported in tumors, where they are thought to result in loss of function [8].

The findings of Yan et al. provide the most directly relevant genomic context for the present case. In their whole-exome sequencing study of 93 individuals with pineal cysts and 21 resected pineal cyst tissues, the authors analyzed pineal cysts under both dominant and recessive inheritance assumptions and identified overrepresentation of selected germline variants, including an OR4D9 variant under the dominant model and a homozygous STIM2 variant under the recessive model. At the gene level, several recurrently altered genes were highlighted, including POP4, GNGT2, TMEM254, GPHB5, and DLC1. Importantly, however, the study did not establish a single recurrent causal mutation or a clear Mendelian mechanism for pineal cyst formation. Rather, the data suggested genetic heterogeneity and a possible polygenic or pathway-level susceptibility. This interpretation is consistent with our findings: the presence of identical pineal cysts in monozygotic triplets and additional first-degree familial cases supports heritability, but the available data do not yet define a single-gene disorder or a predictable inheritance pattern [29].

The somatic component of the Yan et al. study is particularly relevant to the proposed germline-somatic model in our patient T1. Yan et al. identified 16 somatic variants in 15 genes across 21 pineal cyst tissues, with affected genes involved in transcriptional regulation, epigenetic control, immune modulation, lysosomal biology, and transferase activity. These findings support the concept that acquired alterations in pineal cyst tissue may contribute to cyst formation or maintenance, possibly superimposed on an underlying germline susceptibility. The INO80E frameshift mutation identified in our case is therefore biologically plausible within this broader framework, because INO80E participates in chromatin remodeling, DNA repair, and transcriptional regulation. However, neither HERC2 nor INO80E was among the recurrent or highlighted candidate genes in Yan et al., and our finding should therefore be interpreted as pathway-level convergence rather than direct replication. The low variant allele fractions reported by Yan et al. also emphasize the need for caution, as somatic variants in pineal cyst tissue may reflect marked cellular heterogeneity of the pineal gland and cyst wall rather than a uniform clonal driver [29].

Additional support for a genetic contribution to pineal-region cystic abnormalities comes from studies of heritable retinoblastoma. Gupta et al. reported pineal cysts in 26 of 69 children with retinoblastoma, with a significantly higher frequency in heritable retinoblastoma than in sporadic disease: 19 of 38 patients with heritable retinoblastoma had pineal cysts compared with 3 of 26 patients with sporadic retinoblastoma. Most cysts remained stable, decreased in size, or resolved, suggesting that these lesions were usually benign despite occurring in a genetically predisposed population. This observation is important for the present study because it demonstrates that germline alterations affecting neurodevelopmental or tumor-suppressor pathways may be associated with benign pineal cyst formation, without implying that the cysts themselves are neoplastic. Recent molecular studies of pineoblastoma further show that malignant pineal parenchymal tumors are genetically and epigenetically distinct lesions, with recurrent alterations involving pathways such as RB1, DICER1, DROSHA, and OTX2. Thus, while genetic susceptibility is biologically plausible in pineal-region pathology, benign pineal cysts should remain clearly distinguished from pineal parenchymal tumors [12, 14].

The variable clinical phenotype in our triplets is also consistent with prior literature suggesting that cyst formation and symptom generation may be partially separable processes. Arenas et al. reported a markedly increased prevalence of pineal cysts among adults who stutter and proposed that pineal cysts may represent either a biomarker of shared neurodevelopmental vulnerability or, less likely, a direct contributor through local mass effect. Similarly, De Filippo et al. found pineal cysts in 12.2% of girls undergoing MRI for early onset of puberty, but reported no significant differences in auxological, hormonal, or pelvic ultrasound parameters between girls with and without pineal cysts, and no unfavorable cyst evolution during follow-up. These studies support the view that pineal cysts may occur in association with developmental or genetically influenced conditions, while often remaining clinically silent. In the present triplets, all three siblings had identical 14-mm cysts, yet only one required surgery, one had limited symptoms, and one was asymptomatic. This discordance suggests that the genetic or developmental factors responsible for cyst formation may differ from the factors that determine symptom burden, which may depend on local anatomy, venous outflow, tectal compression, CSF dynamics, or individual neurobiological susceptibility [5, 11].

## Limitations

This study is limited by its case report design and small sample size, which restrict generalizability and prevent causal inference. The presence of identical pineal cysts in monozygotic triplets is suggestive but does not confirm a genetic etiology. A key limitation is the shared intrauterine and early-life environment, which makes it difficult to separate genetic from environmental influences. The absence of comparator groups, such as dizygotic siblings or unrelated controls, further limits interpretation.

Family assessment was incomplete, as imaging and genetic evaluation were not systematically performed across the extended pedigree. Potential recall bias and limited clinical data from relatives may have influenced the assessment of familial clustering. Genetic analysis was performed in a single individual using whole-exome sequencing, which does not capture non-coding regions or structural variants. In addition, the identified variants were not functionally validated, limiting conclusions regarding their biological significance.

The variability in clinical presentation among affected individuals also limit the ability to establish consistent genotype–phenotype relationships. Larger, prospectively collected cohorts with comprehensive genetic and clinical characterization are needed to clarify the potential hereditary basis of pineal cysts.

## Conclusion

This study offers insight into the role genetics may play in pineal cyst formation, as highlighted by the rare observation of identical cysts in monozygotic triplets and the presence of similar findings among relatives. Although causality cannot be established based on this case alone, the findings contribute meaningfully to the literature and highlights the importance of continued research in this area. These preliminary insights may eventually support the development of personalized diagnostic and management strategies for patients with a genetic predisposition to pineal cysts.

## Data Availability

No datasets were generated or analysed during the current study.

## Abbreviations

CSF: Cerebrospinal fluid
DNA: Deoxyribonucleic acid
EPS: Encapsulated PostScript
GRCh38: Genome Reference Consortium Human Build 38
HERC2: HECT and RLD domain-containing E3 ubiquitin protein ligase 2
Hg38: Human genome assembly 38
IGV: Integrative Genomics Viewer
INO80E: INO80 complex subunit E
MRI: Magnetic resonance imaging
PC: Pineal cyst
RLD: Regulator of chromosome condensation 1-like domain
T1: Patient triplet 1
T2: Patient triplet 2
T3: Patient triplet 3
WES: Whole-exome sequencing
